# An Emotional Analysis Method for the Analysis of Cognitive and Psychological Factors in the Change of Second Language Learning Model of Chinese Mainland Students in the Post-epidemic Era

**DOI:** 10.3389/fpsyg.2022.819855

**Published:** 2022-07-26

**Authors:** Gang Xie, Xiaona Wang

**Affiliations:** ^1^School of Foreign Language Education, Jilin University, Changchun, China; ^2^School of Arts, Agricultural Technology of Jilin, Jilin City, China

**Keywords:** post-epidemic era, second language learning model, change of learning model, cognitive and psychological factors, an emotional analysis method, statistical analysis

## Abstract

Since the sudden outbreak of the coronavirus disease 2019 (COVID-19) epidemic in 2020, the second language learning patterns of students in mainland China have encountered new challenges that have had a psychological impact on mainland Chinese students. The epidemic has not only inconvenienced students’ normal second language learning but also greatly affected the second language learning patterns of mainland Chinese students. In the post-epidemic era, more and more students are becoming accustomed to studying and learning a second language online. The level of informatization of second language learning patterns of students in mainland China has increased significantly. This study first analyses the mechanisms of change in second language learning patterns and further analyses the influence of knowledge background on the perception of second language learning patterns on this basis. To design the influencing factors of second language learning patterns, a questionnaire was used to investigate the influence of knowledge background on the perception of second language learning patterns. The survey was conducted on students who were learning a second language in mainland China. Then, the survey data were statistically analyzed. In analyzing the influence of effect on second language learning behaviors of students in mainland China, observed variables were designed, including observed variables of affective factors and learning behaviors. After that, the findings of the experiment were summarized based on the results of the questionnaire survey, and the positive influence of emotional factors on second language learning behaviors of mainland Chinese students in the post-development era was concluded.

## Introduction

While the new crown virus epidemic has caused many inconveniences to school teaching, it has also brought profound changes and impacts to the second language learning model of mainland Chinese students (Xiuqin and Yingli, 2020; Mohsen and Mahdi, 2021). Under the influence of the epidemic, the ecology of the second language learning model for mainland Chinese students has undergone tremendous changes: students have developed the habit of learning a second language online, teachers’ traditional teaching thinking has also changed, and the development of informatization of the second language learning model for students in mainland China has also taken a big step forward. First, there have been profound changes in students’ habits of learning a second language in mainland China. Almost all students have formed the habit of learning a second language online as they are forced to leave school to continue their studies at home due to the epidemic prevention and control (Calafato, 2021). This habit is reflected in the following aspects: the goal of second language learning can be achieved without relying on the carrier of the classroom, good learning results can be achieved without relying on traditional learning methods, and the goal of second language learning can be achieved through autonomous learning without relying on the supervision of teachers to a certain extent (Mohsen and Mahdi, 2021). Therefore, judging from students’ performance in learning a second language, the epidemic has had a profound impact on the teaching ecology of second language learning for students in mainland China. Second, teachers’ teaching thinking has undergone profound changes. During the epidemic, all learning models were basically converted to online, and all teaching activities were carried out based on modern information technology. The teaching thinking of teachers was very different from the thinking of traditional teaching methods (Barrios and Hayes-Harb, 2020; Bigelow et al., 2020). This difference is mainly manifested in the following: teaching content materials must conform to the characteristics of online teaching methods, the way of interaction between teachers and students in the process of distance teaching has changed, and the way of feedback on teaching effects has also changed (Wahbeh et al., 2021). Therefore, the teaching thinking of teachers has also changed with the changes in the teaching ecology and has shown the characteristics of the Internet. Finally, the degree of informatization of the second language learning model for mainland Chinese students has been greatly improved. Driven by the epidemic, the online learning model of the second language for students on the Chinese mainland has been popularized, greatly improving the informatization level of the second language learning. On the one hand, the informationization of the second language learning model of students in mainland China breaks the limitation of physical space teaching and provides rich learning resources and teaching methods for second language learning (Martin, 2020). On the other hand, the informatization of students’ second language learning model in mainland China has greatly improved the informatization ability and professional quality of second language teachers (Hsieh, 2020; Havron, 2021). Therefore, affected by the epidemic, the degree of informatization of the second language learning model for mainland Chinese students has greatly increased, which in turn improves the overall teaching level of the second language learning for mainland Chinese students.

This article consists of the following sections: the first section is the introduction, which introduces the significance and importance of this study; the second section examines the mechanism of change in the second language learning model; the third section analyses the influence of knowledge background on the perception of the second language learning model; the fourth section analyses the influence of emotion on learning behavior, and the fifth section is the conclusion.

## Mechanism of Change in the Second Language Learning Model

Current studies generally simplify the generation of behavior into a driving model of “need-motive-attitude-intention-behavior” (Cong and Chen, 2021; Thomas et al., 2021). Need is the reflection in the brain of an individual’s objective needs of the internal and external environment, including material, psychological, and spiritual needs. Motivation is the internal power that impels an individual to engage in some kind of activity (Hou and Aryadoust, 2021), the intrinsic condition that causes motive is need, and the extrinsic condition is inducement. Attitude is people’s evaluation of things based on their own morality and values. Intention is the behavioral tendency of the individual toward the object of the attitude, that is, the state of preparation for the behavior; behavior refers to the external activities of a person under the influence of subjective and objective factors and is an overall process of action (Tabata-Sandom et al., 2020).

A need arises when an individual feels a lack of something materially, psychologically, or spiritually, and a need creates motivation to satisfy it (Gong et al., 2021). However, individuals do not immediately carry out the behavior but evaluate the behavior to be carried out and form an attitude toward the behavior (Gessinger et al., 2021). Iglesias et al. (2021) believed that adding intention as an intermediary variable determines whether a behavior occurs.

**FIGURE 1:**
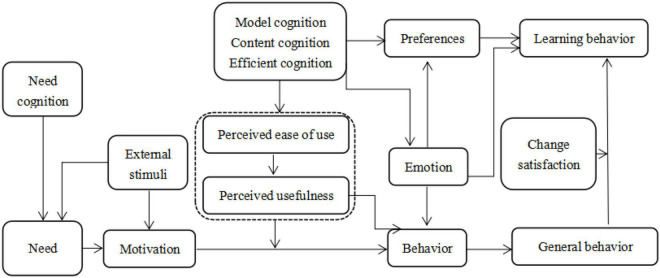
The formation mechanism of learning behavior of those whose second language learning model is changed.

When the individual realizes the need and converts the need into the motivation for learning under the stimulation of external incentives, the individual, therefore, becomes the subject of the second language learning model. Next, the changed subjects will conduct cognition and understanding of the second language learning model, evaluate the usefulness and ease of use of the second language learning model, and form the attitude of the second language learning model after obtaining the results. At the same time, through the cognition and experience of the second language learning model, the changed subjects can generate certain emotions, which are positive and negative. When the positive emotions rise to a certain height, the person being changed will have learning intentions, and the intentions will ultimately determine the occurrence of the changed behavior (Loingsigh and Mozzon-Mcpherson, 2020; Lou and Noels, 2020). After the behavior occurs, the subject will evaluate the degree of pleasure in the second language learning model, thus producing the satisfaction of the subject (Martín-Monje and Borthwick, 2021). However, the changed subject is not a completely rational decision-maker. The behavior of the changed subject is affected by their own emotions to some extent, and they have preference for the second language learning model. Only when the changed subject has preference for the second language learning model emotionally, the behavior can be maintained. The formation mechanism of learning behavior of those whose second language learning model is changed is shown in Figure 1.

According to the above theoretical analysis, the formation mechanism of the learning behavior of the person whose second language learning model is changed is obtained (Figure 1). This article proposes the hypothesis that the emotions and preferences of the changed subjects have a positive effect on the learning behavior of the changed subjects. However, for the second language learning model, the changed subject’s cognition of the second language learning model will affect the changed subject’s preference and emotions for the second language learning model, thereby affecting the learning behavior of the person being changed; hence, it is necessary to understand what factors affect the changed subject’s perception of the second language learning model.

The following research will first analyze the influence of the changed subject’s own factors on the cognition of the second language learning model, and then construct the emotional factors and the changed subject’s learning structure model and the personal preference and the changed subject’s learning structure model on this basis, and then conduct a questionnaire to investigate, and finally verify the hypothesis and make recommendations.

## An Analysis of the Influence of Knowledge Background on Second Language Learning Model Cognition

Due to the different factors of the altered subjects, the altered subjects’ perceptions of the second language learning model also differ, which affects the altered subjects’ preferences and emotions toward the second language learning model. This study focuses on analyzing the influence of subjects with different knowledge and experience backgrounds on the perception of second language learning models, i.e., observing and analyzing the differences in the perception of the content of second language learning models by subjects with different knowledge backgrounds.

### Design of Influencing Factors

In this study, the knowledge background of the change in the second language learning model of Chinese mainland students in the post-epidemic era is divided into four dimensions: professional background, academic education, retrieval knowledge, and second language learning experience (Xu, 2020); according to this, the changed subjects were grouped to analyze the differences between groups. Specific user groups were divided as follows:

*Professional background* is divided into science and engineering, humanities, and other three groups.
*Academic education* is divided into undergraduate (junior and senior students), master’s students, and doctoral students.
*Retrieval knowledge* is divided into proficient, skilled, and general groups, according to the degree of mastery of the changed subjects.
*The second language learning experience* is based on the frequency of second language learning, which is divided into four groups, namely, at least once a week, once a half month, once a month, and at most once a month.

As for the determination of website cognitive content, this study divides it into three parts: pattern cognition, content cognition, and efficiency cognition. The specific contents of each part are as follows: (1) Pattern cognition includes the cognition of the function, content, and process of the second language learning model of Chinese mainland students in the post-epidemic era. (2) Content cognition includes the cognition of the information comprehensiveness, content types, updating of teaching methods, and teaching quality of second language learning patterns of Chinese mainland students in the post-epidemic era. 3. The cognition of efficiency includes the cognition of the efficiency of changing the second language learning model of Chinese mainland students in the post-epidemic era.

### The Questionnaire Survey

The subjects of this questionnaire survey are all mainland Chinese students who are learning a second language, mainly college students. A total of 200 questionnaires were distributed and 192 valid questionnaires were recovered. The effective questionnaire response rate was 96.00%. A summary of the basic information of the survey objects in the valid questionnaire is shown in Table 1.

**TABLE 1:** Summary of the basic information of the survey objects in the valid questionnaire.

Survey item	Description	Number of samples	Proportion (%)
Professional background	Science and engineering	102	53.13
	Humanities	56	29.17
	Other	34	17.70
Academic education	Undergraduate (junior and senior students)	130	67.71
	Master students	47	24.48
	Doctoral students	15	7.81
Retrieval knowledge	Proficient	43	22.40
	Skilled	69	35.94
	General	80	41.66
The second language learning experience	At least once a week	68	35.42
	Once a half month	62	32.29
	Once a month	48	25.00
	At most once a month	14	7.29

### Statistical Analysis of Data

The cognition experiment of the changed subjects mainly analyses the difference in the cognition of the change of the second language learning model of Chinese students in the post-epidemic era by the changed subjects with different second language learning knowledge experience. In this study, SPSS20.0 software (IBM, Chicago, IL, United States) is used to collect statistics on the data, and a one-way ANOVA method is used. It is generally believed that the *P*-value of the changed person variable is less than 0.1 after the cognitive univariate analysis of the change in the second language learning model of mainland Chinese students in the post-epidemic era. It shows that this variable has a significant impact on the change of cognition of the second language learning model of Chinese students in the post-epidemic era.

First, the impact of the second language learning knowledge experience of the changed subject on the cognition of the second language learning model of Chinese students in the post-epidemic era is analyzed. The analysis results are shown in Table 2.

**TABLE 2:** Variance test of the impact of second language learning knowledge and experience on the cognitive impact of the second language learning model of Chinese students in the post-epidemic era.

Individual characteristic variables	Basic understanding of the second language learning model	The role of second language learning model for cognition	Perception of acceptance of second language learning model
Professional background	0.002	0.004	0.413
Academic education	0.318	0.523	0.732
Retrieval knowledge	0.926	0.027	0.527
The second language learning experience	0.957	0.186	0.134

According to Table 2, it can be seen that (1) the changed subjects of different professional backgrounds have significant differences in the basic cognition of the second language learning model and the cognitive evaluation of the role of the second language learning model and (2) there are significant differences in the cognitive evaluation of the effect of different levels of retrieval knowledge on the second language learning model.

Second, the influence of the knowledge and experience of the changed participants on the content cognition of the second language learning model is analyzed. The results are shown in Table 3.

**TABLE 3:** Test of variance of score of influence of knowledge experience on content cognition of second language learning model.

Individual characteristic variables	Comprehensiveness of content	Second language learning quality cognition	Real-time cognition of content update	Contrast cognition with offline second language learning models
Professional background	0.185	0.005	0.165	0.115
Academic education	0.563	0.415	0.530	0.862
Retrieval knowledge	0.421	0.352	0.481	0.034
The second language learning experience	0.436	0.647	0.836	0.047

According to Table 3, it can be seen that (1) changed subjects with different professional backgrounds have significant differences in the timeliness of the information update of the second language learning model and the cognitive evaluation of the comparison with the offline second language learning model (Meskill et al., 2020), (2) there are significant differences in the cognitive evaluation of the teaching quality of the second language learning model by the changed subjects with different second language learning experiences, and (3) there are significant differences in the cognitive evaluation of the effect of the second language learning model among those who have different levels of retrieval knowledge.

Finally, the impact of the knowledge and experience of the changed subjects on the cognitive effect of the change of the second language learning model of Chinese students in the post-epidemic era is analyzed. The results are shown in Table 4.

**TABLE 4:** Variance test of the cognitive impact of the knowledge and experience of the changed subjects on the efficiency of the change of the second language learning model of Chinese students in the post-epidemic era.

Individual characteristic variables	Perception of total efficiency	Recognition of personalized learning efficiency	Cognition of teaching effect
Professional background	0.075	0.218	0.117
Academic education	0.263	0.327	0.962
Retrieval knowledge	0.561	0.036	0.132
The second language learning experience	0.051	0.401	0.034

According to Table 4, it can be seen that (1) there are significant differences in the cognitive evaluation of the efficiency of the change in second language learning model of mainland Chinese students in the post-epidemic era among the subjects with different professional backgrounds and different second language learning experiences; (2) the subjects with different levels of retrieval knowledge had significant differences in their cognitive evaluation of the personalized learning efficiency of the change of second language learning model in the post-epidemic era; and (3) there are significant differences in cognitive evaluation of teaching effect among the subjects with different second language learning experiences (Hsieh, 2020).

## Analysis of the Influence of Emotion on Learning Behavior

Based on the above theoretical research, the author puts forward a mechanism model of the effect of affective factors on the learning behavior of the changed subjects and puts forward hypothesis H1: Affective analysis has a positive effect on the learning behavior of the changed subjects (Warren, 2020). The main purposes of emotion analysis are as follows: (1) Analyze the hypothesis of the influence of sentimental factors on the learning behavior of the changed subject and examine the effect of emotion analysis on the learning behavior of the changed person in general (Ebadi et al., 2021); (2) Comparative analysis of emotion analysis and the weights of the observed variables of the learning behavior of the changed person.

Emotional factor is the inner psychological reflection of the tendency of people and objective things to be good or bad in the activities of people. Between people, a good emotional relationship is established, and it can make people feel affectionate. With a sense of cordiality, the mutual attraction is great, and the influence of each other is also great. Conversely, without the establishment of a good emotional relationship, it will cause a certain psychological distance between the two sides, and the psychological distance is a psychological repulsive force; confrontation will produce a negative influence. So, students must inject their own situational factors when learning a second language, but in the post-epidemic era, students have more time for online classes; hence, it is more important to adjust their emotional perceptions in time and try not to influence their learning behavior.

### Design of Observed Variables

#### Design of Observed Variables for Emotion Factors

This study divides the emotion factors into the mood, satisfaction, and surprise of the changed subject after learning. In fact, the design and content of the second language learning model have an impact on the above-mentioned emotions of users. When the changed subject is learning a second language, the optimized second language learning model after emotion analysis can make the changed subject feel relaxed and happy in the second language learning process and enhance the second language learning experience (Gong et al., 2021). If the content and teaching effect provided by the second language learning exceed the expectation of the subject, the subject will feel surprised and be more patient in the process of second language learning, so as to ensure the effect of second language learning. Affected by the epidemic, students’ demand for the informationization degree of the second language learning model has increased, and the second language learning model supported by informationization technology can break the limitation of physical space teaching, and improve the changed students’ trust in the second language learning model, and significantly affect their emotions (Zheng et al., 2020).

#### Design of Observed Variables for Learning Behavior

In this study, the measurement items of second language learning behavior change of students in mainland China are divided into learning behavior and learning attitude. The observed variables of learning behavior were divided into percentage of second language learning and percentage of second language application. The observational variables of learning attitude were divided into learning intention, preferred future learning intention, and fault tolerance. The observed variables of emotion factors and learning behaviors are shown in Table 5.

**TABLE 5:** The observed variables of emotion factors and learning behaviors.

Structure variables	Observation variable
Emotion factors	Change experience mood
	Change satisfaction
	Change the degree of surprise
The changed subject’s learning behavior	The number of sessions in a second language learning class
	Percentage of study time
	The ability to learn
	Study notes
	Learning effect

**FIGURE 2:**
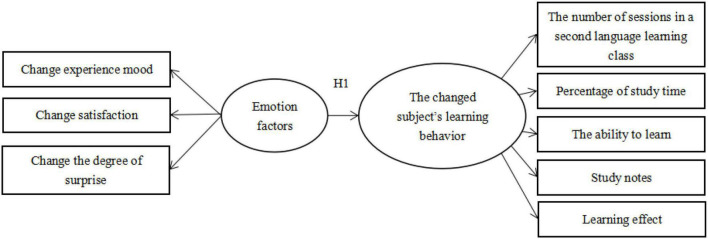
The conceptual model of affective factors influencing the learning behavior of the changed students.

The conceptual model of affective factors influencing the learning behavior of the changed students is finally formed, as shown in Figure 2.

### The Questionnaire Survey

This questionnaire is divided into three parts. The first part is the basic information of the interviewees. The second part is an investigation of the learning behavior of the people whose learning model is changed, which includes learning behavior and learning attitude. Learning behavior is measured by the frequency of learning, percentage of learning time, and learning ability in second language learning classes. Learning attitude is measured by learning experience, learning effect, and other indicators (Ez-Zaouia et al., 2020; Zheng and Tian, 2020). The third part is the measurement of emotional factors, including the changed mood of the subject, the degree of satisfaction with the change of the second language learning model of mainland Chinese students in the post-epidemic era, and the degree of surprise to the change of the second language learning model of mainland Chinese students in the post-epidemic era.

The respondents of this questionnaire are all Chinese mainland students, mainly college students, who are learning a second language. A total of 150 questionnaires were distributed, and 144 valid questionnaires were recovered.

### Statistical Analysis of Data

SPSS20.0 statistical software is used to complete the statistics of basic data. The reliability and validity test results of structural variables are shown in Table 6.

**TABLE 6:** The reliability and validity test results of structural variables.

Structure variables	Number of observed variables	AVE	Cronbach’s α
Emotion factors	3	0.812	0.925
The changed subject’s learning behavior	5	0.827	0.913

Table 6 shows that Cronbach’s α values reach 0.925 and 0.913, which means that the questions show good internal consistency. In addition, the AVE value is significantly higher than the threshold value of 0.7. It shows that latent variables have strong convergent validity. It can be seen that the measure items of the questionnaire have high reliability. The weight summary table of observed variables is shown in Table 7.

**TABLE 7:** The weight summary table of observed variables.

Structure variables	Observation variable	Interpretation of observed variables	Weight of observed variable
Emotion factors	Change experience mood	The mood of the subject after experiencing the change of the second language learning model	0.326
	Change satisfaction	The satisfaction of the changed subjects after experiencing the change of second language learning model	0.354
	Change the degree of surprise	How surprised they are after experiencing the change in their second language learning model	0.320
The changed subject’s learning behavior	The number of sessions in a second language learning class	The number of times the subject studied in a second language learning class	0.218
	Percentage of study time	Percentage of second language learning in total learning time	0.179
	The ability to learn	Changes in second language learning ability	0.282
	Study notes	Changes in second language learning experience	0.114
	Learning effect	Changes in the effect of second language learning	0.207

From Table 7, it can be known that the weights of the observed variables of the affective factors are not much different, and they are all around 0.320. Among them, the weight of the emotion of the changed subject for the second language learning experience is relatively high, which shows that the emotion of the changed subject for the second language learning model change experience has a considerable influence on the cognitive effect of the second language learning model change. The weight of learning ability is more important than the weight of learning experience. In the attitude part, the weight of learning ability is the highest, while the weight of learning experience is the lowest.

### Experimental Conclusion

Based on the above data analysis, the following conclusions can be drawn from the affective analysis. The hypothesis that affective factors have a positive influence on the second language learning behavior of students in mainland China in the post-emergency era is verified, and affective factors have a significant positive influence on the learning effect; among the affective factors, experiencing mood has a greater influence on the effect of the changed students, which indicates that the change in the second language learning model of students in mainland China in the post-emergency era has a great influence on the mood of the changed students in the process of learning the second language. Therefore, our suggestion is to let students have a correct view of learning, whether in online classes or in classrooms. First, we teachers should observe students’ emotional changes more before the class and actively make good emotional guidance, and second, if students are at home by themselves, then teachers should communicate more with students to eliminate students’ loneliness. The last thing is to make students’ homework not too much.

## Conclusion

Understanding the cognitive and psychological processes of second language learners is helpful for teachers to adjust foreign language teaching methods and improve the efficiency of foreign language learning (Silvia, 2020). In the post-epidemic era, computer technology provides a good means for second language teachers to develop more attractive software and apply it in teaching (Lochlainn et al., 2021). The online learning model created by online education platforms and software in the post-epidemic era has exerted a subtle influence on students. Online learning is gradually attracting mainland Chinese students with its strong atmosphere of the times, convenient implementation methods, and massive knowledge treasures (Zeng et al., 2020). This second language learning model may play a greater role in the future learning of mainland Chinese students. In order to help students achieve better online learning of the second language learning, teachers still need to continue to study online teaching method to improve student’s comprehensive ability and strive to increase online interaction and teaching platform for the school, to help students form a good habit of learning a second language, and at the same time, the students also need to continuously strengthen their self-cultivation to improve the consciousness of learning (Obermeier and Elgort, 2020).

In the post-epidemic era, the knowledge of mainland Chinese students has a significant impact on the second language learning model’s model cognition and content cognition, while the knowledge and experience of the changed person have a significant impact on the second language learning model’s service cognition. Therefore, strengthening the cognition of the changed subject’s second language learning model can promote the changed subject’s learning behavior. Emotion factors have a significant impact on the learning behavior of the changed second language learning model, among which the mood of the changed second language learning experience has a greater impact on loyalty. When the changed subject has positive feelings toward the second language learning model, the willingness of the changed person to choose the second language learning model next time can be improved. Personal preference has a significant impact on the learning behavior of the changed learners. The convenience of the interface design of the changed learners and the variety of the content of the changed learners have a greater impact on their learning behavior. Based on the above research conclusions, the second language learning model for students in mainland China in the post-epidemic era can improve the learning behavior of the changed learners from the following aspects.

In the future, we should focus more on students’ behavioral cognition and affective cognition because we know from the above studies that these two cognitions can influence students’ learning behaviors, and we can also add more factors in future designs so that we can evaluate students’ learning behaviors more completely, regardless of future epidemics.

## Data Availability Statement

The original contributions presented in the study are included in the article/supplementary material, further inquiries can be directed to the corresponding authors.

## Ethics Statement

The studies involving human participants were reviewed and approved by Jilin University. The patients/participants provided their written informed consent to participate in this study.

## Author Contributions

GX collected and analyzed data. XW contributed to research and edited the article. Both authors contributed to the article and approved the submitted version.

## Conflict of Interest

The authors declare that the research was conducted in the absence of any commercial or financial relationships that could be construed as a potential conflict of interest.

## Publisher’s Note

All claims expressed in this article are solely those of the authors and do not necessarily represent those of their affiliated organizations, or those of the publisher, the editors and the reviewers. Any product that may be evaluated in this article, or claim that may be made by its manufacturer, is not guaranteed or endorsed by the publisher.
